# Association between delirium and statin use in patients with congestive heart failure: a retrospective propensity score-weighted analysis

**DOI:** 10.3389/fnagi.2023.1184298

**Published:** 2023-06-20

**Authors:** Jiangling Xia, Leilei Wang, Nannan Zhang, Hongyu Xu

**Affiliations:** ^1^Department of Anesthesiology, Zibo Central Hospital, Zibo, Shandong, China; ^2^School of Architecture and Engineering, Zibo Vocational Institute, Zibo, Shandong, China; ^3^Department of Anesthesiology, Affiliated Hospital of Qingdao University Medical College, Qingdao, Shandong, China

**Keywords:** delirium, mortality, congestive heart failure, statin, propensity analysis

## Abstract

**Background:**

The relationship between statin use and delirium remains controversial; therefore, we aimed to study the association between statin exposure and delirium and in-hospital mortality in patients with congestive heart failure.

**Methods:**

In this retrospective study, patients with congestive heart failure were identified from the Medical Information Mart for Intensive Care database. The primary exposure variable was statin use 3 days after admission to the intensive care unit, and the primary outcome measure was the presence of delirium. The secondary outcome measure was in-hospital mortality. Since the cohort study was retrospective, we used inverse probability weighting derived from the propensity score to balance various variables.

**Results:**

Of 8,396 patients, 5,446 (65%) were statin users. Before matching, the prevalence of delirium was 12.5% and that of in-hospital mortality was 11.8% in patients with congestive heart failure. Statin use was significantly negatively correlated with delirium, with an odds ratio of 0.76 (95% confidence interval: [0.66–0.87]; *P* < 0.001) in the inverse probability weighting cohort and in-hospital mortality of 0.66 (95% confidence interval: [0.58–0.75]; *P* < 0.001).

**Conclusion:**

Statins administered in the intensive care unit can significantly reduce the incidence of delirium and in-hospital mortality in patients with congestive heart failure.

## 1. Introduction

Delirium manifests as a state of acute attention, cognitive impairment, and mental disorders that may be related to physiological disorders (Oh et al., 2017). Delirium is common in both young and older patients with acute heart failure (AHF), and its occurrence in the intensive care unit (ICU) is related to both short- and long-term mortality (Iwata et al., 2020; Han et al., 2022). Uthamalingam et al. (2011) found that in patients with congestive heart failure (CHF), delirium increased 30- and 90-day readmission and short-term mortality rates. Matthew et al. (Duprey et al., 2020) found that patients with or without delirium before admission to the ICU might have different disease trajectories upon admission. As delirium treatments are limited, routine screening for sensory impairments and cognitive status of older patients for the highest risk of in-hospital delirium has been proven important, preventive interventions could be relevant and effective in preventing delirium in vulnerable populations (Correale et al., 2020; Monacelli et al., 2022). Recently, contradictory evidence about the role of statins in preventing delirium has been established. Some studies have revealed that statins can reduce the occurrence of delirium, including that in the ICU and postoperative delirium (Mather et al., 2017; Lee et al., 2018). In contrast, other studies have reported that the effect of statins on delirium is related to the severity of the disease and not to its occurrence (An et al., 2023; Chang et al., 2023). However, most studies have shown that statins significantly reduce all-cause mortality (Orkaby et al., 2020; Ng et al., 2022) and mortality in patients with cancer (Orkaby et al., 2020; Kang et al., 2021).

To date, no studies have examined the effect of oral statins on delirium in patients with CHF. We used the Medical Information Mart for Intensive Care-IV (MIMIC-IV) database (Johnson et al., 2023) to investigate the relationship between statins and delirium and in-hospital mortality in the ICU in patients with CHF. In this study, we performed a propensity score-weighted analysis. The propensity score matching (PSM) method was used to appropriately adjust for the confounding factors and reduce the impact of these deviations and confounding variables to make a reasonable comparison between the statin-exposed and non–statin-exposed groups.

## 2. Materials and methods

### 2.1. Data source

This retrospective cohort study was based on the MIMIC-IV database (version 2.2), which comprised data from patients admitted to the ICU of the Beth Israel Deaconess Medical Center in Boston, Massachusetts between 2008 and 2019. One author (JLX) obtained access to the database and was responsible for data extraction. The establishment of the MIMIC-IV database was approved by the institutional review boards of both Beth Israel Deaconess Medical Center and Massachusetts Institute of Technology Affiliates. The requirement for informed consent was waived because the data of all patients in the database were anonymized.

### 2.2. Study population and data extraction

We included all patients who were first admitted to the ICU with CHF from the MIMIC-IV database (version 2.2). To extract the raw data from the database, we excluded (i) patients with dementia, (ii) patients younger than 18 years old, and (iii) patients without Confusion Assessment Method for the ICU estimation using Navicat Premium (version 16.0). The extracted data included demographics, laboratory test results, vital signs, comorbidities, and administered drugs. The following demographic information was extracted: age, sex, and length of hospitalization. Vital sign data such as systolic blood pressure (SBP), diastolic blood pressure (DBP), heart rate (HR), respiratory rate (RR), and oxygenated hemoglobin saturation (SpO_2_) were collected. Data regarding comorbidities including diabetes, chronic pulmonary disease, peripheral vascular disease, malignant cancer, cerebrovascular disease, myocardial infarction (MI), liver disease, and renal disease were extracted. Laboratory data including the white blood cell (WBC) and platelet (PLT) counts; hematocrit (HCT) value; hemoglobin (HGB), blood glucose, creatinine, blood urea nitrogen (BUN), potassium, sodium, and calcium levels; and anion gap were collected. We also extracted details of whether the patient underwent mechanical ventilation (VENT). Details of the use of vasoactive drugs, norepinephrine, vasopressin, and epinephrine or administration of angiotensin-converting enzyme inhibitors/angiotensin receptor blockers (ACEIs/ARBs), statins, diuretics, and β-blockers 3 days after admission to the ICU were extracted. The Simplified Acute Physiology Score (II) and the Charlson comorbidity index, which represents the severity of the disease, were also included. In total, 6% of calcium levels were missing and <0.1% of the other indicator levels were missing among the laboratory test results. Because these missing covariate data are continuous variables, we replaced them with their means. This allowed us to use the data collected from an incomplete dataset.

### 2.3. Medication exposure

Regarding statin use, we defined patients with records of 3 days of statin use after admission to the ICU as statin-exposed and others as non–statin-exposed. We searched drug ILIKE “statin” and NOT ILIKE “nystatin,” “mycostatin,” “imipenem-cilastatin,” “pentostatin,” and “sandostatin” in Navicat Premium (version 16.0). The medication prescriptions were recorded in the MIMIC-IV (version 2.2) “mimic-hospital, prescription” table.

### 2.4. Outcomes

The primary outcome was the occurrence of delirium during the ICU stay. The secondary outcome was the in-hospital mortality rate. The Confusion Assessment Method for the Intensive Care Unit (CAM-ICU) method was used to evaluate delirium in patients (Ely et al., 2001).

### 2.5. Statistical analyses

Patient characteristics are described overall and by group (statin-exposed and non–statin-exposed). The measured data are expressed as mean (standard deviation) or median (interquartile interval) according to whether they were normally distributed. A one-way analysis of variance or the Kruskal–Wallis H-test was performed depending on whether the data were normally distributed. Categorical variables are expressed as percentages and were assessed using chi-square tests. PSM was used to adjust for confounders between the non–statin-exposed and statin-exposed groups. The following prognostic variables related to the outcome at a *P*-value of < 0.2 in a univariate analysis (Supplementary Table 1) were included in the propensity score: age; sex; HCT; WBC; anion gap; hemoglobin, creatinine, blood urea nitrogen, potassium, and calcium levels; peripheral vascular, cerebrovascular, and liver diseases; SBP, DBP, HR, RR, and SpO_2_ on the first day of admission; ACEI/ARB and β-blocker administration after admission to the ICU; and mechanical ventilation. Moreover, norepinephrine, vasopressin, and epinephrine use and diuretics were forced into the PSM. The variables included in the in-hospital mortality analysis are shown in Supplementary Table 2. Inverse probability treatment weighting and overlap weighting with propensity scores were also used. The two groups were matched at a 1:1 ratio with a caliper width of 0.2. The standardized mean difference was used to examine the degree of PSM. The R software package (http://www.R-project.org, The R Foundation) and Free Statistics software version 1.7 were used to perform all statistical analyses. Statistical differences were considered significant at a *P*-value of < 0.05.

## 3. Results

### 3.1. Baseline characteristics

**Figure 1:**
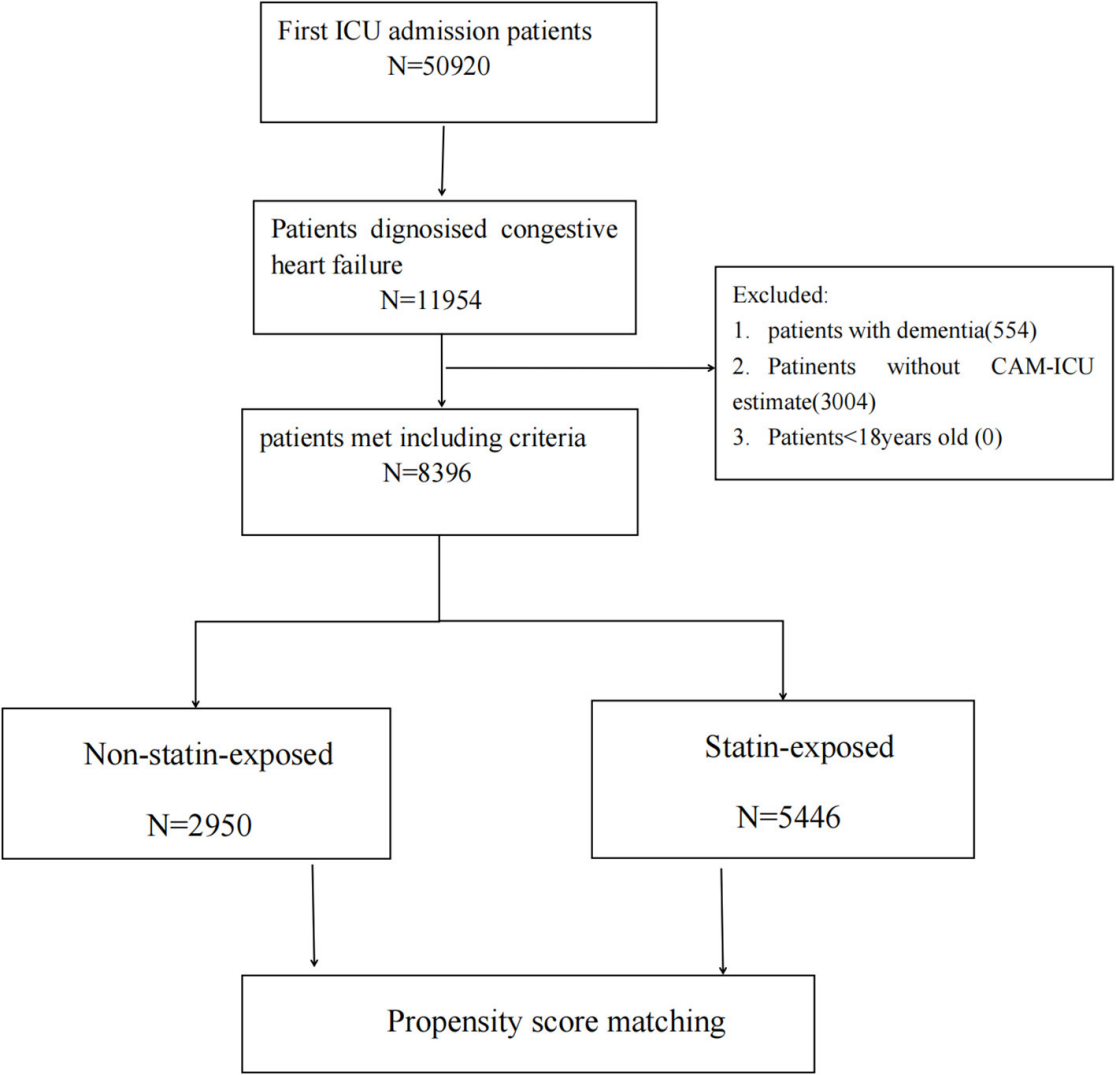
Flowchart of cohort selection.

Of the 50,920 patients who were first admitted to the ICU and included in the MIMIC-IV database, 8,396 patients with CHF were evaluated using the Confusion Assessment Method for the ICU method. The patient selection process is shown in Figure 1. Table 1 summarizes the characteristics of the statin-exposed and non–statin-exposed groups. A total of 5,446 (65%) patients were exposed to statins. The median age of the patients was 75 years (range: 64–83 years), and 56.8% were men. The total incidence of delirium was 12.5% (1047/8396) and that of in-hospital mortality was 11.8% (991/8396). Patients in the statin-exposed group had a higher age, a higher rate of diabetes, and chronic pulmonary, peripheral vascular, renal, and cerebrovascular diseases. Moreover, they received more ACEI and β-blockers than did those in the non–statin-exposed group (all *P* < 0.05); however, they had lower delirium (10.6–15.9%), length of hospital stay (8.1–8.8 days), and in-hospital mortality (9.7–15.7%) (all *P* < 0.05).

**Table 1:** Baseline characteristics.

**Variables**		**Non–statin-exposed**	**Statin-exposed**	***p***
	**Total (*****n*** = **8,396)**	**(*****n*** = **2,950)**	**(*****n*** = **5,446)**	
Age (year)	75.0 (64.0, 83.0)	72.0 (60.0, 84.0)	75.0 (67.0, 83.0)	< 0.001
Gender (male%)	4,772 (56.8)	1,520 (51.5)	3,252 (59.7)	< 0.001
HR (beats/min)	84.2 ± 16.1	87.3 ± 17.6	82.6 ± 15.1	< 0.001
RR (beats/min)	19.9 ± 3.7	20.3 ± 4.0	19.6 ± 3.5	< 0.001
SBP (mmHg)	116.6 ± 16.5	115.7 ± 17.0	117.1 ± 16.2	< 0.001
DBP (mmHg)	61.7 ± 11.3	63.0 ± 11.8	61.0 ± 11.0	< 0.001
SpO_2_ (%)	96.6 ± 2.1	96.4 ± 2.3	96.6 ± 2.0	< 0.001
HCT (%)	30.7 ± 6.8	31.1 ± 7.2	30.5 ± 6.6	< 0.001
HGB (g/L)	10.0 ± 2.3	10.1 ± 2.4	9.9 ± 2.2	0.001
PLT (10^∧^9/L)	183.3 ± 89.8	183.6 ± 98.9	183.1 ± 84.5	0.831
WBC (10^∧^9/L)	14.4 ± 10.5	14.7 ± 11.9	14.2 ± 9.7	0.03
Anion gap (mmol/L)	17.0 ± 4.7	17.4 ± 5.2	16.8 ± 4.4	< 0.001
BUN (mmol/L)	35.8 ± 25.8	36.5 ± 27.4	35.4 ± 24.9	0.062
Calcium (mg/dL)	8.3 ± 0.8	8.2 ± 0.8	8.3 ± 0.7	< 0.001
Creatinine (mg/dL)	1.9 ± 1.8	1.9 ± 1.9	1.9 ± 1.7	0.911
Glucose (mg/dL)	122.8 ± 43.8	117.3 ± 40.8	125.7 ± 45.1	< 0.001
Sodium (mmol/L)	139.4 ± 4.7	139.3 ± 5.0	139.4 ± 4.5	0.461
Potassium (mmol/L)	4.7 ± 0.9	4.7 ± 0.9	4.7 ± 0.8	0.747
MI (%)	2,839 (33.8)	447 (15.2)	2,392 (43.9)	< 0.001
Peripheral vascular disease (%)	1,421 (16.9)	350 (11.9)	1,071 (19.7)	< 0.001
Cerebrovascular disease (%)	1,146 (13.6)	337 (11.4)	809 (14.9)	< 0.001
Chronic pulmonary disease (%)	3,034 (36.1)	1,082 (36.7)	1,952 (35.8)	0.447
Renal disease (%)	3,141 (37.4)	885 (30)	2,256 (41.4)	< 0.001
Diabetes (%)	3,404 (40.5)	815 (27.6)	2,589 (47.5)	< 0.001
Cancer (%)	870 (10.4)	388 (13.2)	482 (8.9)	< 0.001
Liver disease (%)	779 (9.3)	448 (15.2)	331 (6.1)	< 0.001
Epinephrine (%)	423 (5.0)	137 (4.6)	286 (5.3)	0.224
Norepinephrine (%)	1,553 (18.5)	516 (17.5)	1,037 (19)	0.081
Vasopressin (%)	923 (11.0)	314 (10.6)	609 (11.2)	0.451
Diuretics	6,531(77.8)	2,213(75)	4,318(79.3)	< 0.001
β_blocker (%)	5,433 (64.7)	1,615 (54.7)	3,818 (70.1)	< 0.001
ACEI/ARB (%)	1,930 (23.0)	468 (15.9)	1,462 (26.8)	< 0.001
VENT (%)	2,439 (21.0)	878 (29.8)	1,661 (28.7)	0.289
CCI	7.0 (6.0, 9.0)	7.0 (5.0, 8.0)	8.0 (6.0, 9.0)	< 0.001
SAPS II	37.0 (30.0, 46.0)	38.0 (30.0, 47.0)	37.0 (30.0, 45.0)	0.007
Delirium (%)	1,047 (12.5)	468 (15.9)	579 (10.6)	< 0.001
Hospital-mortality (%)	991 (11.8)	462 (15.7)	529 (9.7)	< 0.001
Los hospital (day)	8.3 (5.2, 13.8)	8.8 (5.2, 14.6)	8.1 (5.1, 13.5)	0.006

Data are presented as mean (sd), medians [interquartile ranges], or numbers (percentages).

HR, heart rate; RR, respiratory rate; SBP, systolic blood pressure; DBP, diastolic blood pressure; HCT, hematocrit; HGB, hemoglobin.WBC, white blood cell count; PLT, platelets; BUN, blood urea nitrogen; MI, myocardial infarction; ACEIs/ARBs, angiotensin-converting enzyme inhibitors/angiotensin receptor blockers; SAPS II, Simplified Acute Physiology Score; VENT, Mechanical ventilation; CCI, Charlson Comorbidity Index; Los, length of stay.

### 3.2. PSM analysis

**Figure 2:**
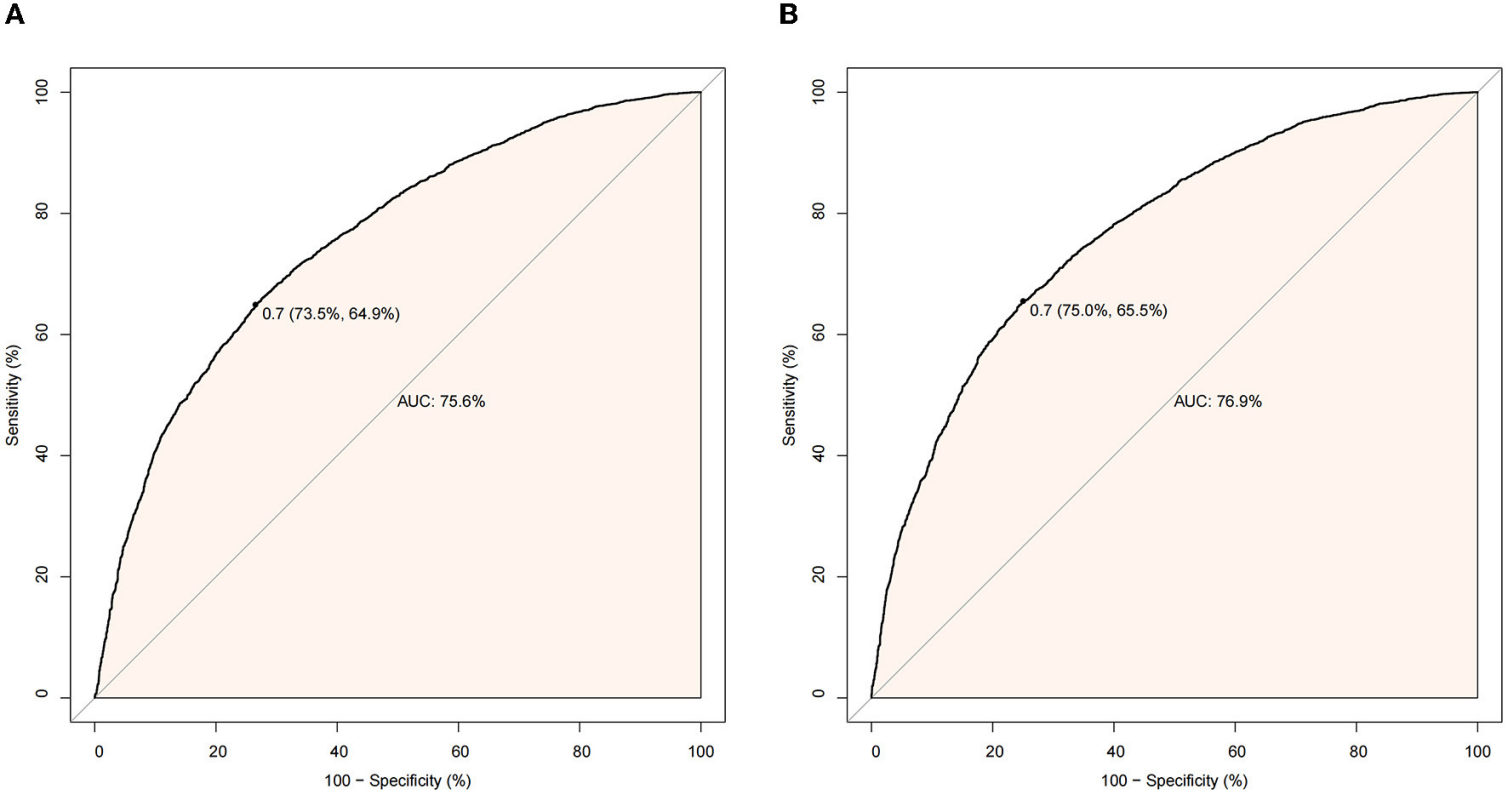
**(A)** Receiver operating characteristic (ROC) curve for delirium. **(B)** Receiver operating characteristic (ROC) curve for in-hospital mortality.

We used PSM to balance the baseline characteristics. After matching, 2,429 and 2,331 patients with delirium and in-hospital mortality, respectively, were included in each group. The standardized mean difference of all covariates after matching was <0.1, indicating a sufficient balance after matching (Tables 2, 3). Furthermore, we reported variables in the subject operating characteristic curves for delirium (Figure 2A) and in-hospital mortality (Figure 2B). The area under the curve was calculated to assess the relationship between statin use and delirium (75.6%) and in-hospital mortality (76.9%).

**Table 2:** Imbalance of patient characteristics before and after propensity score matching in the assessment of delirium.

**Variables**	**Unmatched**			**SMD**	**Matched**			**SMD**
	**Non–statin-exposed**	**Statin-exposed**	**SMD**	**0.1**	**Non–statin-exposed**	**Statin-exposed**	**SMD**	**0.1**
	2,950	5,446			2,429	2,429		
Age (year)	70.29 (16.34)	74.24 (11.71)	0.278	>0.1	72.82 (14.73)	72.80 (12.50)	0.002	< 0.1
Gender (male)	1,520 (51.5)	3,252 (59.7)	0.165	>0.1	1,271 (52.3)	1,291 (53.1)	0.016	< 0.1
HR (beats/min)	87.34 (17.58)	82.57 (15.06)	0.292	>0.1	85.21 (16.65)	85.09 (16.22)	0.007	< 0.1
RR(beats/min)	20.29 (3.98)	19.64 (3.48)	0.175	>0.1	19.94 (3.74)	19.89 (3.65)	0.013	< 0.1
SBP (mmHg)	115.70 (16.95)	117.12 (16.22)	0.086	< 0.1	116.53 (17.04)	116.90 (16.49)	0.022	< 0.1
DBP (mmHg)	63.04 (11.82)	61.00 (11.01)	0.179	>0.1	62.35 (11.51)	62.28 (11.44)	0.006	< 0.1
SpO_2_ (%)	96.43 (2.33)	96.65 (2.03)	0.099	< 0.1	96.51 (2.23)	96.53 (2.11)	0.009	< 0.1
HCT (%)	31.15 (7.20)	30.52 (6.56)	0.092	< 0.1	30.88 (6.90)	30.94 (6.77)	0.009	< 0.1
HGB (g/L)	10.11 (2.37)	9.94 (2.22)	0.072	< 0.1	10.03 (2.27)	10.05 (2.26)	0.01	< 0.1
WBC (10^∧^9/L)	14.69 (11.86)	14.17 (9.69)	0.048	< 0.1	14.29 (11.05)	14.37 (10.10)	0.007	< 0.1
Anion gap (mmol/L)	17.41 (5.22)	16.76 (4.44)	0.134	>0.1	16.98 (4.78)	16.91 (4.66)	0.014	< 0.1
BUN (mmol/L)	36.51 (27.36)	35.42 (24.85)	0.042	< 0.1	36.08 (26.68)	36.17 (26.19)	0.003	< 0.1
Calcium (mg/dL)	8.19 (0.84)	8.31 (0.72)	0.157	>0.1	8.25 (0.81)	8.26 (0.76)	0.014	< 0.1
Creatinine (mg/dL)	1.86 (1.85)	1.86 (1.72)	0.003	< 0.1	1.83 (1.83)	1.82 (1.70)	0.009	< 0.1
Glucose (mg/dL)	117.29 (40.83)	125.73 (45.08)	0.196	>0.1	119.46 (41.29)	120.46 (40.67)	0.024	< 0.1
Sodium (mmol/L)	139.34 (5.04)	139.42 (4.46)	0.017	< 0.1	139.39 (4.86)	139.48 (4.72)	0.019	< 0.1
Potassium (mmol/L)	4.70 (0.89)	4.71 (0.84)	0.007	< 0.1	4.69 (0.88)	4.68 (0.85)	0.005	< 0.1
MI	447 (15.2)	2,392 (43.9)	0.665	>0.1	436 (17.9)	451 (18.6)	0.016	< 0.1
Peripheral vascular disease	350 (11.9)	1,071 (19.7)	0.215	>0.1	336 (13.8)	330 (13.6)	0.007	< 0.1
Cerebrovascular disease	337 (11.4)	809 (14.9)	0.102	>0.1	298 (12.3)	328 (13.5)	0.037	< 0.1
Chronic pulmonary disease	1,082 (36.7)	1,952 (35.8)	0.017	< 0.1	904 (37.2)	916 (37.7)	0.01	< 0.1
Liver disease	448 (15.2)	331 (6.1)	0.299	>0.1	237 (9.8)	247 (10.2)	0.014	< 0.1
CCI	6.82 (2.74)	7.71 (2.35)	0.352	>0.1	7.13 (2.69)	7.17 (2.34)	0.018	< 0.1
SAPS II	39.55 (14.20)	38.50 (12.04)	0.08	< 0.1	39.18 (12.94)	39.25 (13.16)	0.006	< 0.1
Epinephrine	137 (4.6)	286 (5.3)	0.028	< 0.1	115 (4.7)	113 (4.7)	0.004	< 0.1
Norepinephrine	516 (17.5)	1,037 (19.0)	0.04	< 0.1	433 (17.8)	405 (16.7)	0.031	< 0.1
Vasopressin	314 (10.6)	609 (11.2)	0.017	< 0.1	260 (10.7)	253 (10.4)	0.009	< 0.1
β_blocker	1,615 (54.7)	3,818 (70.1)	0.321	>0.1	1,449 (59.7)	1,457 (60.0)	0.007	< 0.1
ACEI/ARB	468 (15.9)	1,462 (26.8)	0.27	>0.1	437 (18.0)	437 (18.0)	< 0.001	< 0.1
VENT	878 (29.8)	1,561 (28.7)	0.024	< 0.1	702 (28.9)	718 (29.6)	0.014	< 0.1
Diuretics	2,213 (75.0)	4,318 (79.3)	0.102	>0.1	1,853 (76.3)	1,869 (76.9)	0.016	< 0.1

An absolute MSD of <10% was considered to support the assumption of a balance between the groups. SMD, standardized mean differences. Data are presented as mean [SD] or as numbers (percentages).

HR, heart rate; RR, respiratory rate; SBP, systolic blood pressure; DBP, diastolic blood pressure; HCT, hematocrit; HGB, hemoglobin; WBC, white blood cell count; BUN, blood urea nitrogen; MI, myocardial infarction; SAPS II, Simplified Acute Physiology Score; CCI, Charlson Comorbidity Index; ACEIs/ARBs, angiotensin-converting enzyme inhibitors/angiotensin receptor blockers; VENT, mechanical ventilation.

**Table 3:** Imbalance of patient characteristics before and after propensity score matching in the assessment of in-hospital mortality.

**Variables**	**Unmatched**			**SMD 0.1**	**Matched**			**SMD**
	**Non–statin-exposed**	**Statin-exposed**	**SMD**		**Non–statin-exposed**	**Statin-exposed**	**SMD**	**0.1**
	2,950	5,446			2,331	2,331		
Age (years)	70.29 (16.34)	74.24 (11.71)	0.278	>0.1	73.04 (14.59)	72.88 (12.51)	0.011	< 0.1
Gender (male)	1,520 (51.5)	3,252 (59.7)	0.165	>0.1	1,241 (53.2)	1,254 (53.8)	0.011	< 0.1
HR (beats/min)	87.34 (17.58)	82.57 (15.06)	0.292	>0.1	85.15 (16.59)	85.27 (16.26)	0.007	< 0.1
RR (beats/min)	20.29 (3.98)	19.64 (3.48)	0.175	>0.1	19.93 (3.73)	19.91 (3.64)	0.005	< 0.1
SBP (mmHg)	115.70 (16.95)	117.12 (16.22)	0.086	< 0.1	116.49 (16.94)	117.02 (16.56)	0.032	< 0.1
DBP (mmHg)	63.04 (11.82)	61.00 (11.01)	0.179	>0.1	62.24 (11.49)	62.44 (11.45)	0.017	< 0.1
SpO_2_ (%)	96.43 (2.33)	96.65 (2.03)	0.099	< 0.1	96.49 (2.24)	96.55 (2.09)	0.026	< 0.1
HCT (%)	31.15 (7.20)	30.52 (6.56)	0.092	< 0.1	30.92 (6.87)	30.92 (6.83)	< 0.001	< 0.1
HGB(g/L)	10.11 (2.37)	9.94 (2.22)	0.072	< 0.1	10.04 (2.27)	10.04 (2.28)	0.001	< 0.1
WBC (10^∧^9/L)	14.69 (11.86)	14.17 (9.69)	0.048	< 0.1	14.52 (12.02)	14.45 (10.35)	0.006	< 0.1
Anion gap (mmol/L)	17.41 (5.22)	16.76 (4.44)	0.134	>0.1	17.02 (4.82)	16.94 (4.70)	0.016	< 0.1
BUN (mmol/L)	36.51 (27.36)	35.42 (24.85)	0.042	< 0.1	35.78 (25.77)	35.71 (26.13)	0.003	< 0.1
Calcium (mg/dL)	8.19 (0.84)	8.31 (0.72)	0.157	>0.1	8.26 (0.81)	8.26 (0.76)	0.004	< 0.1
Creatinine (mg/dL)	1.86 (1.85)	1.86 (1.72)	0.003	< 0.1	1.83 (1.77)	1.81 (1.80)	0.01	< 0.1
Glucose (mg/dL)	117.29 (40.83)	125.73 (45.08)	0.196	>0.1	119.89 (42.35)	120.75 (40.48)	0.021	< 0.1
Potassium (mmol/L)	4.70 (0.89)	4.71 (0.84)	0.007	< 0.1	4.69 (0.88)	4.68 (0.86)	0.003	< 0.1
MI	447 (15.2)	2,392 (43.9)	0.665	>0.1	438 (18.8)	468 (20.1)	0.033	< 0.1
Peripheral vascular disease	350 (11.9)	1,071 (19.7)	0.215	>0.1	324 (13.9)	315 (13.5)	0.011	< 0.1
Cerebrovascular disease	337 (11.4)	809 (14.9)	0.102	>0.1	292 (12.5)	317 (13.6)	0.032	< 0.1
Chronic pulmonary disease	1,082 (36.7)	1,952 (35.8)	0.017	< 0.1	865 (37.1)	891 (38.2)	0.023	< 0.1
Liver disease	448 (15.2)	331 (6.1)	0.299	>0.1	239 (10.3)	237 (10.2)	0.003	< 0.1
Renal disease	885 (30.0)	2,256 (41.4)	0.24	>0.1	779 (33.4)	766 (32.9)	0.012	< 0.1
Cancer	388 (13.2)	482 (8.9)	0.138	>0.1	264 (11.3)	286 (12.3)	0.029	< 0.1
Diabetes	815 (27.6)	2,589 (47.5)	0.42	>0.1	763 (32.7)	770 (33.0)	0.006	< 0.1
CCI	6.82 (2.74)	7.71 (2.35)	0.352	>0.1	7.09 (2.64)	7.13 (2.43)	0.018	< 0.1
SAPSII	39.55 (14.20)	38.50 (12.04)	0.08	< 0.1	39.23 (13.16)	38.99 (13.02)	0.018	< 0.1
Epinephrine	137 (4.6)	286 (5.3)	0.028	< 0.1	109 (4.7)	111 (4.8)	0.004	< 0.1
Norepinephrine	516 (17.5)	1,037 (19.0)	0.04	< 0.1	411 (17.6)	387 (16.6)	0.027	< 0.1
Vasopressin	314 (10.6)	609 (11.2)	0.017	< 0.1	250 (10.7)	242 (10.4)	0.011	< 0.1
β_blocker	1,615 (54.7)	3,818 (70.1)	0.321	>0.1	1,422 (61.0)	1,423 (61.0)	0.001	< 0.1
ACEI/ARB	468 (15.9)	1,462 (26.8)	0.27	>0.1	424 (18.2)	434 (18.6)	0.011	< 0.1
Diuretics	2,213 (75.0)	4,318 (79.3)	0.102	>0.1	1,786 (76.6)	1,792 (76.9)	0.006	< 0.1

An absolute MSD of < 10% was considered to support the assumption of a balance between the groups. SMD, standardized mean differences. Data are presented as mean [SD] or as numbers (percentages).

HR, heart rate; RR, respiratory rate; SBP, systolic blood pressure; DBP, diastolic blood pressure; HCT, hematocrit; HGB, hemoglobin; WBC, white blood cell count; BUN, blood urea nitrogen; MI, myocardial infarction; SAPS II, Simplified Acute Physiology Score; CCI, Charlson Comorbidity Index; ACEIs/ARBs, angiotensin-converting enzyme inhibitors/angiotensin receptor blockers.

### 3.3. Association between statin exposure and its outcomes

Before matching, regression analysis showed that statin exposure was significantly associated with delirium at an odds ratio of 0.63 (95% confidence interval: 0.55–0.72, *P* < 0.001) (Table 4). After inverse probability weighting, the risk of delirium remained significantly associated with statin exposure at an odds ratio of 0.76 (95% confidence interval: 0.66–0.87, *P* < 0.001). Before matching, the risk of in-hospital mortality was significantly related to statin exposure at an odds ratio of 0.58 (95% confidence interval: 0.51–0.66, *P* < 0.001). After inverse probability weighting, the risk of in-hospital mortality remained significantly associated with statin exposure at an odds ratio of 0.66 (95% confidence interval: 0.58–0.75, *P* < 0.001). The results were similar to those obtained with the PSM model. Moreover, we used overlap weighting with propensity scores, and the results remained robust.

**Table 4:** Association between statin use and delirium and in-hospital mortality in the crude analysis, multivariable analysis, PSM, IPTW, and PS-OW.

**Analysis**	**Item**	**Delirium**		**In-hospital mortality**	
		**OR-95CI**	***P***	**OR-95CI**	***P***
Crude analysis	Statin-exposed **vs**. non–statin-exposed	0.63 (0.55–0.72)	< 0.001	0.58 (0.51–0.66)	< 0.001
Multivariable analysis		0.78 (0.67–0.9)	0.001	0.7(0.58–0.82)	< 0.001
PSM		0.82(0.69–0.97)	0.02	0.64 (0.53–0.76)	< 0.001
IPTW		0.76 (0.66–0.87)	< 0.001	0.66 (0.58–0.75)	< 0.001
PS-OW		0.8 (0.65–0.99)	0.036	0.72 (0.58–0.89)	0.003

OR, odds ratio; CI, confidence interval; PSM, propensity score matching; IPTW, Inverse probability treatment weighting; PS-OW, overlap weighting with propensity score.

### 3.4. Subgroup analyses

**Figure 3:**
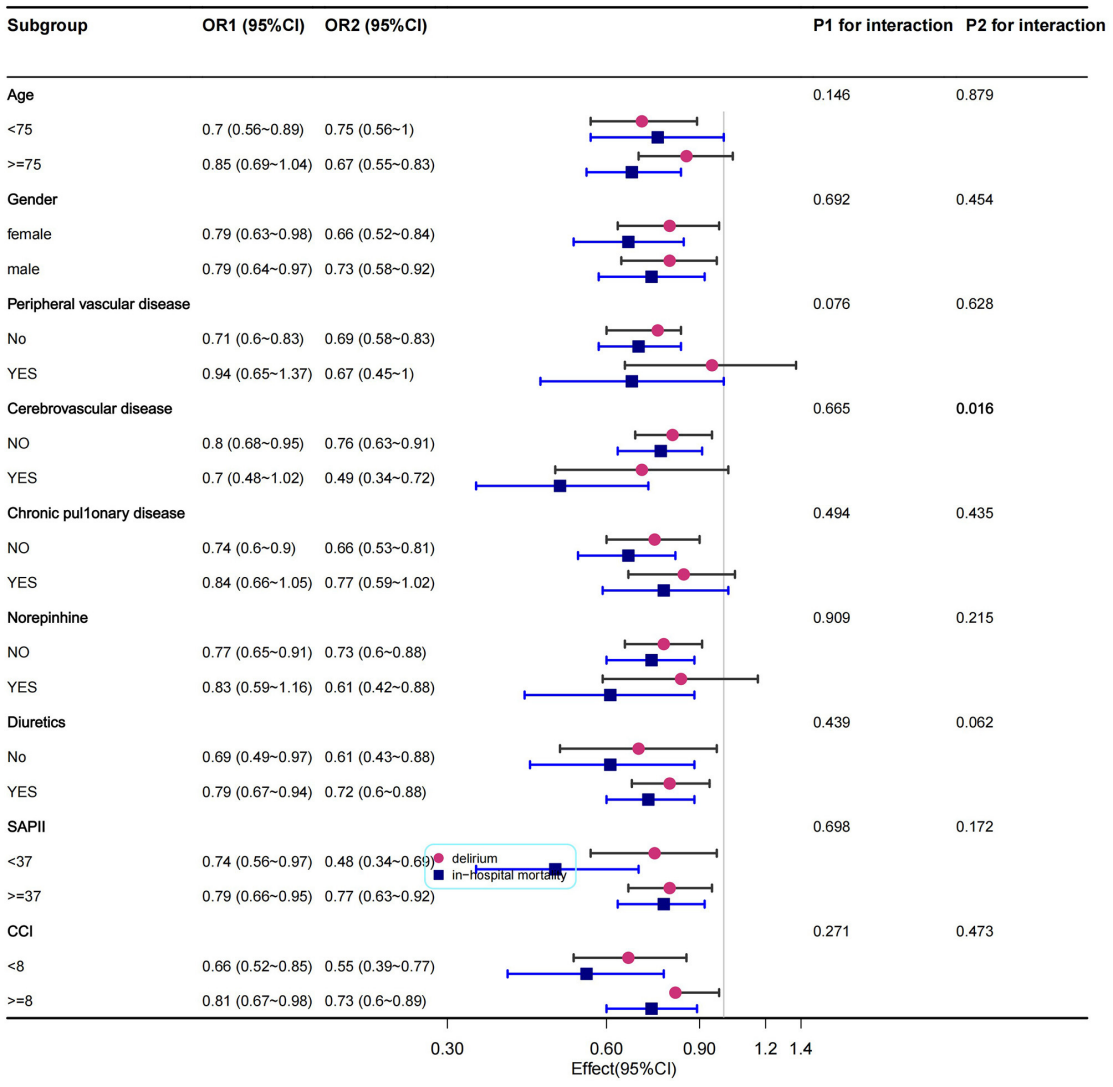
Subgroup analysis of the relationship between statin exposure and delirium and in-hospital mortality in patients with CHF.

As shown in Figure 3 (the original form is in Supplementary Tables 3, 4), only cerebrovascular disease showed an interaction between statin exposure and in-hospital mortality (*P* = 0.016). The *P*-value for the interactions in the other subgroups showed no interaction with delirium and in-hospital mortality, and the subgroup analyses were adjusted for the PSM variables.

## 4. Discussion

In this observational study involving patients with CHF, we used PSM and found that statin administration after ICU admission was significantly associated with a reduced risk of delirium and in-hospital mortality. To the best of our knowledge, this is the first observational study to investigate the association between statin exposure and delirium and in-hospital mortality in patients with CHF.

A systematic review and meta-analysis showed that statin therapy did not affect delirium status in critically ill and cardiac surgery patients (Vallabhajosyula et al., 2017). In a randomized controlled trial, Sohrevardi et al. found that atorvastatin administration at a dose of 40 mg/day reduced delirium in patients in the ICU (Sohrevardi et al., 2021). Our findings were consistent with those of Mather et al. (2017), who suggested that statin administration reduced ICU delirium. Another cohort study confirmed that statin use could significantly reduce delirium among critically ill patients in the ICU (Morandi et al., 2014). Page et al. (2014) found that statin use prior to the night of admission was an independent predictor of the absence of delirium. We excluded patients with dementia, which was previously shown to be highly associated with delirium (Fong and Inouye, 2022; Lieberman et al., 2022).

The high incidence of delirium in patients with CHF is related to its specific pathophysiology; HF is often associated with atherosclerosis, hypotension, and hypoxia, which may cause inadequate cerebral perfusion and is associated with delirium (Hori et al., 2014; Soh et al., 2017). Statins have been found to decrease coronary artery disease by reducing atheroma volume and stabilizing atheromatous plaques to prevent the formation of new atherosclerotic lesions, resulting in a lower incidence of HF. Moreover, they reduce the risk of HF in the medium to long term (Lee et al., 2019). Therefore, a significant proportion of patients with coronary artery disease receive statins. Many recent studies have found that premedication with statins reduced mortality in patients in ICU with acute kidney injury, sepsis, and coronavirus disease 2019 (Chinaeke et al., 2021; Tu et al., 2021; Zuin et al., 2021; Lao et al., 2022). Although the exact mechanism remains unclear, statins play an important role in antioxidative stress, anti-inflammatory and anti-cerebral vasospasm effects, and platelet aggregation inhibition (Chen J. et al., 2020). In basic experiments, statins have been found to ameliorate brain edema and early brain injury in rabbits via neuroprotection (Chen J.-H. et al., 2020). Simultaneously, one study found that atorvastatin relieved cerebral vasospasm and mediated structural and functional remodeling of the vascular endothelial cells (Chen et al., 2018). This suggested that statins prevent delirium and mortality.

This study has some limitations. First, baseline level data before admission in the MIMIC-IV database might be incomplete, which might have affected delirium according to the following aspects: preoperative cognitive status, psychiatric history, and educational level. Second, our study did not include long-term prognostic outcomes of delirium in statin users with CHF. Therefore, future prospective studies are required. Third, this was a retrospective study. Although PSM was used to control confounding factors, residual confounders could not be completely excluded. Finally, we cannot be sure whether patients received statins after or before being admitted to the ICU and whether the long-term preventive effect of statins was better although some studies have concluded this (Orkaby et al., 2020).

## 5. Conclusion

In this retrospective analysis, we confirmed that statin use in the ICU was significantly associated with reduced delirium and in-hospital mortality in patients with CHF.

## Data availability statement

The original contributions presented in the study are included in the article/Supplementary material, further inquiries can be directed to the corresponding author.

## Ethics statement

Ethical review and approval was not required for the study on human participants in accordance with the local legislation and institutional requirements. The patients/participants provided their written informed consent to participate in this study.

## Author contributions

JX designed the study and wrote the manuscript. LW modified the manuscript. HX analyzed the manuscript. NZ reviewed the statistical analyses. All authors read and approved the final manuscript.
