# Supplementary material for: Association between delirium and statin use in patients with congestive heart failure: a retrospective propensity score-weighted analysis
Source: Front Aging Neurosci. 2023 Jun 20;15:1184298. doi: 10.3389/fnagi.2023.1184298 (PMC10318247; doi:10.3389/fnagi.2023.1184298)
Supplement: Supplementary file 1 [file Data_Sheet_1.docx]

Supplementary Material

Association ‎ Between Delirium and Statin Use in Patients With ‎‎Congestive Heart Failure: A Retrospective Propensity ‎‎Score‑Matching Analysis

Jiangling Xia^1^ Hongyu Xu^1*^ Nannan Zhang^2^

1 Department of Anesthesiology,Zibo Central Hospital, Zibo,Shandong,China

2 Department of Anesthesiology,Affiliated Hospital of Qingdao University Medical College,Qingdao,Shandong,China

* Correspondence:Department of Anesthesiology,Zibo Central Hospital, Zi Bo,Shan Dong,China
Corresponding Author:Hongyu Xu
E-mail:xuhy009@163.com

# Supplementary Tables

**Supplementary Table 1.**univariate regression analysis for delirium

| Variable | OR_95CI | P_value |
| --- | --- | --- |
| age | 1 (1~1.01) | **0.121** |
| gender | 0.87 (0.76~0.99) | **0.032** |
| HR | 1.01 (1~1.01) | **<0.001** |
| RR | 1.02 (1.01~1.04) | **0.011** |
| SBP | 0.99 (0.99~1) | **0.015** |
| DBP | 0.98 (0.98~0.99) | **<0.001** |
| SpO_2_ | 1.06 (1.03~1.1) | **<0.001** |
| HCT | 1.01 (1~1.02) | **0.177** |
| HGB | 1.03 (1~1.06) | **0.067** |
| PLT | 1 (1~1) | 0.814 |
| WBC | 1.01 (1~1.01) | **<0.001** |
| aniongap | 1.04 (1.03~1.05) | **<0.001** |
| bun | 1.01 (1~1.01) | **<0.001** |
| calcium | 0.73 (0.68~0.79) | **<0.001** |
| creatinine | 1.05 (1.01~1.08) | **0.005** |
| glucose | 1 (1~1) | **0.18** |
| sodium | 1.03 (1.02~1.05) | **<0.001** |
| potassium | 1.05 (0.98~1.13) | **0.18** |
| MI | 0.74 (0.64~0.85) | **<0.001** |
| peripheral vascular disease | 1.17 (0.99~1.38) | **0.067** |
| cerebrovascular disease | 1.33 (1.11~1.58) | **0.001** |
| chronic pul1onary disease | 1.4 (1.22~1.59) | **<0.001** |
| renal disease | 1.03 (0.91~1.18) | 0.618 |
| Liver disease | 2.33(1.77-2.56) | **<0.001** |
| cancer | 0.87 (0.7~1.08) | 0.213 |
| Diabetes | 1 (0.88~1.14) | 0.972 |
| CCI | 1.02 (0.99~1.05) | **0.145** |
| SAPII | 1.04 (1.03~1.04) | **<0.001** |
| Epinephrine | 1.55 (1.2~2.01) | **0.001** |
| Norepinhine | 1.44 (1.23~1.68) | **<0.001** |
| Vasopressin | 1.42 (1.18~1.72) | **<0.001** |
| β_blocker | 0.74 (0.65~0.84) | **<0.001** |
| ACEI/ARB | 0.58 (0.48~0.69) | **<0.001** |
| VENT | 1.25 (1.08~1.43) | **0.002** |
| Deuretics | 1.25 (1.06~1.47) | **<0.001** |

Bolded items are p<0.2.

**Abbreviations**:HR:heart rate .RR: respiratory rate,SBP:Systolic blood pressure.DBP:Diastolic blood pressure.HCT:hematocrit.HGB:Hemoglobin.WBC:white blood Cell count.PLT:platelets.MI:Myocardial infarction,SAPSII: Simplified Acute Physiology Score.CCI:Charlson Comorbidity Index.ACEI/ARB:Angiotensin-converting enzyme inhibitors/angiotensin receptor blockers.VENT:Mechanical ventilation

**Supplementary Table 2.**univariate regression analysis for in-hospital mortality

| Variable | OR_95CI | P_value |
| --- | --- | --- |
| Age | 1.03 (1.03~1.04) | **<0.001** |
| Gender | 0.85 (0.75~0.97) | **0.019** |
| HR | 1.02 (1.01~1.02) | **<0.001** |
| RR | 1.1 (1.09~1.12) | **<0.001** |
| SBP | 0.97 (0.97~0.98) | **<0.001** |
| DBP | 0.98 (0.97~0.98) | **<0.001** |
| SpO2 | 0.9 (0.88~0.93) | **<0.001** |
| HCT | 0.98 (0.97~0.99) | **<0.001** |
| HGB | 0.91 (0.88~0.94) | **<0.001** |
| PLT | 1 (1~1) | 0.525 |
| WBC | 1.02 (1.01~1.02) | **<0.001** |
| Aniongap | 1.11 (1.1~1.12) | **<0.001** |
| Bun | 1.02 (1.01~1.02) | **<0.001** |
| Calcium | 0.7 (0.64~0.76) | **<0.001** |
| Creatinine | 1.12 (1.09~1.15) | **<0.001** |
| Glucose | 1 (1~1) | **0.032** |
| Sodium | 0.99 (0.98~1.01) | 0.339 |
| Potassium | 1.25 (1.17~1.34) | **<0.001** |
| MI | 1.2 (1.05~1.38) | **0.008** |
| Peripheral vascular disease | 1.17 (0.99~1.39) | **0.068** |
| Cerebrovascular disease | 1.87 (1.58~2.21) | **<0.001** |
| Chronic pulmonary disease | 1.13 (0.99~1.29) | **0.08** |
| Liver desease | 2.27 (1.89~2.73) | **<0.001** |
| Renal disease | 1.56 (1.36~1.78) | **<0.001** |
| Cancer | 1.9 (1.58~2.28) | **<0.001** |
| Diabetes | 0.9 (0.79~1.03) | **0.134** |
| CCI | 1.19 (1.16~1.23) | **<0.001** |
| SAPII | 1.07 (1.07~1.08) | **<0.001** |
| Epinephrine | 1.9 (1.48~2.44) | **<0.001** |
| Norepinhine | 1.56 (1.34~1.82) | **<0.001** |
| Vasopressin | 1.75 (1.46~2.1) | **<0.001** |
| β_blocker | 0.36 (0.32~0.42) | **<0.001** |
| ACEI/ARB | 0.26 (0.21~0.33) | **<0.001** |
| VENT | 1.05 (0.9~1.21) | 0.545 |
| Deuretics | 0.8 (0.69~0.93) | **0.005** |

Bolded items are p<0.2.

**Abbreviations**:HR:heart rate .RR: respiratory rate,SBP:Systolic blood pressure.DBP:Diastolic blood pressure.HCT:hematocrit.HGB:Hemoglobin.WBC:white blood Cell count.PLT:platelets.MI:Myocardial infarction,SAPSII: Simplified Acute Physiology Score.CCI:Charlson Comorbidity Index.ACEI/ARB:Angiotensin-converting enzyme inhibitors/angiotensin receptor blockers.VENT:Mechanical ventilation

**Supplementary Table 3.**Subgroup analysis of the relationship between statin exposure and delirium in patients with CHF

| Subgroup | Variable | n.total | n.event_% | crude.OR_95CI | crude.P_value | adj.OR_95CI | adj.P_value | P.for.interaction |
| --- | --- | --- | --- | --- | --- | --- | --- | --- |
| Age<75 |  |  |  |  | 0.146 |  |  |  |
|  | non-statin-exposed | 1611 | 251 (15.6) | 1(Ref) |  | 1 | 1 | 1 |
|  | statin-exposed | 2575 | 234 (9.1) | 0.7 (0.56~0.89) |  | 0.7 | 0.56 | 0.89 |
| Age>=75 |  |  |  |  |  |  |  |  |
|  | non-statin-exposed | 1339 | 217 (16.2) | 1(Ref) |  | 1 | 1 | 1 |
|  | statin-exposed | 2871 | 345 (12) | 0.85 (0.69~1.04) |  | 0.85 | 0.69 | 1.04 |
| Gender(female) |  |  |  |  | 0.692 |  |  |  |
|  | non-statin-exposed | 1430 | 228 (15.9) | 1(Ref) |  | 1 | 1 | 1 |
|  | statin-exposed | 2194 | 256 (11.7) | 0.79 (0.63~0.98) |  | 0.79 | 0.63 | 0.98 |
| Gender(male) |  |  |  |  |  |  |  |  |
|  | non-statin-exposed | 1520 | 240 (15.8) | 1(Ref) |  | 1 | 1 | 1 |
|  | statin-exposed | 3252 | 323 (9.9) | 0.79 (0.64~0.97) |  | 0.79 | 0.64 | 0.97 |
| peripheral vascular disease(NO) |  |  |  |  | 0.076 |  |  |  |
|  | non-statin-exposed | 2600 | 412 (15.8) | 1(Ref) |  | 1 | 1 | 1 |
|  | statin-exposed | 4375 | 437 (10) | 0.75 (0.63~0.88) |  | 0.75 | 0.63 | 0.88 |
| peripheral vascular disease(Yes) |  |  |  |  |  |  |  |  |
|  | non-statin-exposed | 350 | 56 (16) | 1(Ref) |  | 1 | 1 | 1 |
|  | statin-exposed | 1071 | 142 (13.3) | 0.95 (0.65~1.39) |  | 0.95 | 0.65 | 1.39 |
| cerebrovascular disease(NO) |  |  |  |  | 0.665 |  |  |  |
|  | non-statin-exposed | 2613 | 400 (15.3) | 1(Ref) |  | 1 | 1 | 1 |
|  | statin-exposed | 4637 | 471 (10.2) | 0.8 (0.68~0.95) |  | 0.8 | 0.68 | 0.95 |
| cerebrovascular disease(Yes) |  |  |  |  |  |  |  |  |
|  | non-statin-exposed | 337 | 68 (20.2) | 1(Ref) |  | 1 | 1 | 1 |
|  | statin-exposed | 809 | 108 (13.3) | 0.7 (0.48~1.02) |  | 0.7 | 0.48 | 1.02 |
| chronic pul1onary disease(NO) |  |  |  |  | 0.494 |  |  |  |
|  | non-statin-exposed | 1868 | 273 (14.6) | 1(Ref) |  | 1 | 1 | 1 |
|  | statin-exposed | 3494 | 323 (9.2) | 0.74 (0.6~0.9) |  | 0.74 | 0.6 | 0.9 |
| chronic pul1onary disease(Yes) |  |  |  |  |  |  |  |  |
|  | non-statin-exposed | 1082 | 195 (18) | 1(Ref) |  | 1 | 1 | 1 |
|  | statin-exposed | 1952 | 256 (13.1) | 0.84 (0.66~1.05) |  | 0.84 | 0.66 | 1.05 |
| Norepinhine(NO) |  |  |  |  | 0.909 |  |  |  |
|  | non-statin-exposed | 2434 | 359 (14.7) | 1(Ref) |  | 1 | 1 | 1 |
|  | statin-exposed | 4409 | 440 (10) | 0.77 (0.65~0.91) |  | 0.77 | 0.65 | 0.91 |
| Norepinhine(Yes) |  |  |  |  |  |  |  |  |
|  | non-statin-exposed | 516 | 109 (21.1) | 1(Ref) |  | 1 | 1 | 1 |
|  | statin-exposed | 1037 | 139 (13.4) | 0.83 (0.59~1.16) |  | 0.83 | 0.59 | 1.16 |
| Diuretics(NO) |  |  |  |  | 0.439 |  |  |  |
|  | non-statin-exposed | 737 | 106 (14.4) | 1(Ref) |  | 1 | 1 | 1 |
|  | statin-exposed | 1128 | 93 (8.2) | 0.69 (0.49~0.97) |  | 0.69 | 0.49 | 0.97 |
| Diuretics(YES) |  |  |  |  |  |  |  |  |
|  | non-statin-exposed | 2213 | 362 (16.4) | 1(Ref) |  | 1 | 1 | 1 |
|  | statin-exposed | 4318 | 486 (11.3) | 0.79 (0.67~0.94) |  | 0.79 | 0.67 | 0.94 |
| SAPII<7 |  |  |  |  | 0.698 |  |  |  |
|  | non-statin-exposed | 1344 | 134 (10) | 1(Ref) |  | 1 | 1 | 1 |
|  | statin-exposed | 2604 | 167 (6.4) | 0.74 (0.56~0.97) |  | 0.74 | 0.56 | 0.97 |
| SAPII>=7 |  |  |  |  |  |  |  |  |
|  | non-statin-exposed | 1606 | 334 (20.8) | 1(Ref) |  | 1 | 1 | 1 |
|  | statin-exposed | 2842 | 412 (14.5) | 0.79 (0.66~0.95) |  | 0.79 | 0.66 | 0.95 |
| CCI<37 |  |  |  |  | 0.271 |  |  |  |
|  | non-statin-exposed | 1456 | 217 (14.9) | 1(Ref) |  | 1 | 1 | 1 |
|  | statin-exposed | 1795 | 163 (9.1) | 0.66 (0.52~0.85) |  | 0.66 | 0.52 | 0.85 |
| CCI>=37 |  |  |  |  |  |  |  |  |
|  | non-statin-exposed | 1494 | 251 (16.8) | 1(Ref) |  | 1 | 1 | 1 |
|  | statin-exposed | 3651 | 416 (11.4) | 0.81 (0.67~0.98) |  | 0.81 | 0.67 | 0.98 |

**Supplementary Table 4.** Subgroup analysis of the relationship between statin exposure and in-hospital mortality in patients with CHF

| Subgroup | Variable | n.total | n.event_% | crude.OR_95CI | crude.P_value | adj.OR_95CI | adj.P_value | P.for.interaction |
| --- | --- | --- | --- | --- | --- | --- | --- | --- |
| Age<75 |  |  |  |  | 0.879 |  |  |  |
|  | non-statin-exposed | 1611 | 177 (11) | 1(Ref) |  | 1 | 1 | 1 |
|  | statin-exposed | 2575 | 162 (6.3) | 0.75 (0.56~1) |  | 0.75 | 0.56 | 1 |
| Age>=75 |  |  |  |  |  |  |  |  |
|  | non-statin-exposed | 1339 | 285 (21.3) | 1(Ref) |  | 1 | 1 | 1 |
|  | statin-exposed | 2871 | 367 (12.8) | 0.67 (0.55~0.83) |  | 0.67 | 0.55 | 0.83 |
| Gender(female) |  |  |  |  | 0.454 |  |  |  |
|  | non-statin-exposed | 1430 | 239 (16.7) | 1(Ref) |  | 1 | 1 | 1 |
|  | statin-exposed | 2194 | 223 (10.2) | 0.66 (0.52~0.84) |  | 0.66 | 0.52 | 0.84 |
| Gender(male) |  |  |  |  |  |  |  |  |
|  | non-statin-exposed | 1520 | 223 (14.7) | 1(Ref) |  | 1 | 1 | 1 |
|  | statin-exposed | 3252 | 306 (9.4) | 0.73 (0.58~0.92) |  | 0.73 | 0.58 | 0.92 |
| peripheral vascular disease(NO) |  |  |  |  | 0.628 |  |  |  |
|  | non-statin-exposed | 2600 | 395 (15.2) | 1(Ref) |  | 1 | 1 | 1 |
|  | statin-exposed | 4375 | 408 (9.3) | 0.69 (0.58~0.83) |  | 0.69 | 0.58 | 0.83 |
| peripheral vascular disease(Yes) |  |  |  |  |  |  |  |  |
|  | non-statin-exposed | 350 | 67 (19.1) | 1(Ref) |  | 1 | 1 | 1 |
|  | statin-exposed | 1071 | 121 (11.3) | 0.67 (0.45~1) |  | 0.67 | 0.45 | 1 |
| cerebrovascular disease(NO) |  |  |  |  | **0.016** |  |  |  |
|  | non-statin-exposed | 2613 | 369 (14.1) | 1(Ref) |  | 1 | 1 | 1 |
|  | statin-exposed | 4637 | 411 (8.9) | 0.76 (0.63~0.91) |  | 0.76 | 0.63 | 0.91 |
| cerebrovascular disease(Yes) |  |  |  |  |  |  |  |  |
|  | non-statin-exposed | 337 | 93 (27.6) | 1(Ref) |  | 1 | 1 | 1 |
|  | statin-exposed | 809 | 118 (14.6) | 0.49 (0.34~0.72) |  | 0.49 | 0.34 | 0.72 |
| chronic pul1onary disease(NO) |  |  |  |  | 0.435 |  |  |  |
|  | non-statin-exposed | 1868 | 294 (15.7) | 1(Ref) |  | 1 | 1 | 1 |
|  | statin-exposed | 3494 | 314 (9) | 0.66 (0.53~0.81) |  | 0.66 | 0.53 | 0.81 |
| chronic pul1onary disease(Yes) |  |  |  |  |  |  |  |  |
|  | non-statin-exposed | 1082 | 168 (15.5) | 1(Ref) |  | 1 | 1 | 1 |
|  | statin-exposed | 1952 | 215 (11) | 0.77 (0.59~1.02) |  | 0.77 | 0.59 | 1.02 |
| Norepinhine(NO) |  |  |  |  | 0.215 |  |  |  |
|  | non-statin-exposed | 2434 | 346 (14.2) | 1(Ref) |  | 1 | 1 | 1 |
|  | statin-exposed | 4409 | 397 (9) | 0.73 (0.6~0.88) |  | 0.73 | 0.6 | 0.88 |
| Norepinhine(Yes) |  |  |  |  |  |  |  |  |
|  | non-statin-exposed | 516 | 116 (22.5) | 1(Ref) |  | 1 | 1 | 1 |
|  | statin-exposed | 1037 | 132 (12.7) | 0.61 (0.42~0.88) |  | 0.61 | 0.42 | 0.88 |
| Diuretics(NO) |  |  |  |  | 0.062 |  |  |  |
|  | non-statin-exposed | 737 | 152 (20.6) | 1(Ref) |  | 1 | 1 | 1 |
|  | statin-exposed | 1128 | 103 (9.1) | 0.61 (0.43~0.88) |  | 0.61 | 0.43 | 0.88 |
| Diuretics(YES) |  |  |  |  |  |  |  |  |
|  | non-statin-exposed | 2213 | 310 (14) | 1(Ref) |  | 1 | 1 | 1 |
|  | statin-exposed | 4318 | 426 (9.9) | 0.72 (0.6~0.88) |  | 0.72 | 0.6 | 0.88 |
| SAPII<7 |  |  |  |  | 0.172 |  |  |  |
|  | non-statin-exposed | 1344 | 81 (6) | 1(Ref) |  | 1 | 1 | 1 |
|  | statin-exposed | 2604 | 96 (3.7) | 0.48 (0.34~0.69) |  | 0.48 | 0.34 | 0.69 |
| SAPII>=7 |  |  |  |  |  |  |  |  |
|  | non-statin-exposed | 1606 | 381 (23.7) | 1(Ref) |  | 1 | 1 | 1 |
|  | statin-exposed | 2842 | 433 (15.2) | 0.77 (0.63~0.92) |  | 0.77 | 0.63 | 0.92 |
| CCI<37 |  |  |  |  | 0.473 |  |  |  |
|  | non-statin-exposed | 1456 | 138 (9.5) | 1(Ref) |  | 1 | 1 | 1 |
|  | statin-exposed | 1795 | 82 (4.6) | 0.55 (0.39~0.77) |  | 0.55 | 0.39 | 0.77 |
| CCI>=37 |  |  |  |  |  |  |  |  |
|  | non-statin-exposed | 1494 | 324 (21.7) | 1(Ref) |  | 1 | 1 | 1 |
|  | statin-exposed | 3651 | 447 (12.2) | 0.73 (0.6~0.89) |  | 0.73 | 0.6 | 0.89 |
